# Fatty Acid Composition, Oxidative Stability, and Metabolomic Changes in Hickory Nut Oil During Accelerated Oxidation

**DOI:** 10.3390/antiox15030336

**Published:** 2026-03-07

**Authors:** Ziyi Li, Jiahui Liu, Qingqing Gao, Na Zhang, Songyu Geng, Yihong Bao, Qingqi Guo

**Affiliations:** 1 College of Food and Health, Northeast Forestry University, Harbin 150040, China; 2 College of Food Engineering, Harbin University of Commerce, Harbin 150028, China

**Keywords:** hickory nut oil, oxidation stability, metabolomics, metabolic pathway

## Abstract

This study systematically investigated the dynamic changes in fatty acid composition and oxidative stability of hickory nut oil during accelerated oxidation, and characterized the metabolic features associated with lipid oxidation using a metabolomics approach. Accelerated oxidation was conducted using the Schaal oven method. The results showed that the peroxide value (POV) and malondialdehyde (MDA) content reached maximum levels of 121.5 meq/kg and 1.94 μg/mL, while the conjugated diene value (CDV) and carbonyl value (COV) increased to 15.12 and 19.68 meq/kg, respectively. The fatty acid profile exhibited notable changes, with unsaturated fatty acids decreasing from 92.52% to 90.65% and saturated fatty acids increasing from 6.92% to 7.88%. A total of 2026 metabolites were identified, among which fatty acyls and benzenoids were predominant. The oxidation rate remained low during the initial phase (0–10 days) but increased markedly after approximately 25 days, leading to accelerated oxidation and a pronounced decline in oil quality. Pathway enrichment analysis further revealed that α-linolenic acid metabolism was the principal pathway associated with the oxidative process of hickory nut oil.

## 1. Introduction

*Carya cathayensis* (Chinese hickory) is an endemic nut-bearing tree species in China, classified under the genus *Carya* within the family Juglandaceae, exhibits a highly concentrated geographical distribution. As a multifunctional ecological and economic tree species valued for nut production, oil extraction, and timber, it holds considerable potential for integrated utilization. Its nuts are rich in high-quality proteins, abundant lipids, and diverse minerals, conferring notable nutritional and medicinal significance [1]. Among its derivative products, hickory nut oil is distinguished by its abundance of unsaturated fatty acids—primarily oleic and linoleic acids—along with multiple vitamins and minerals, conferring marked nutritional and functional attributes. These components enable lipid regulation, reduce cardiovascular risk, and exert anti-inflammatory and skin health-enhancing effects [2]. However, the high degree of unsaturation renders the oil highly susceptible to oxidative deterioration during processing and storage, as evidenced by rising acid values, off-flavor formation, and progressive quality decline [3]. Consequently, insufficient oxidative stability remains a major bottleneck limiting its broader industrial application [4]. Elucidating the kinetics and formation pathways of oxidation products is therefore crucial for establishing a theoretical basis for quality preservation and enhancing the commercial value and industrial potential of this oil.

Lipid oxidation, driven by oxygen, light exposure, temperature, and metal ions, leads to the depletion of bioactive constituents and essential fatty acids while generating numerous low-molecular-weight compounds with potential toxicity. These oxidation products may induce hepatic damage, impair reproductive function, and cause detrimental changes in flavor, color, and viscosity, thereby compromising oil stability and shortening shelf-life [5]. Additionally, the associated formation of free radicals not only accelerates aging and increases disease susceptibility but may also trigger genetic alterations.

Current investigations on lipid oxidation predominantly rely on chemical and physical analytical techniques, with chemical assays being the most widely applied owing to their simplicity and cost-effectiveness [6]. Nonetheless, these conventional indices capture only partial aspects of oxidative progression and cannot adequately characterize the dynamic transformations within complex lipid matrices, underscoring the need for integrated, multidimensional analytical strategies. Metabolomics—a high-throughput platform enabling comprehensive profiling of small-molecule metabolites—has been widely applied in nutritional research, disease diagnostics, and food science [7,8]. In lipid systems, metabolomics facilitates the identification of oxidation products formed during storage and processing, elucidates their impacts on flavor properties, and enables precise determination of fatty acid compositions. Compared with traditional chemical analyses, it provides a more holistic and temporally resolved understanding of oxidative dynamics. Nevertheless, most LC–MS-based oxidation studies remain largely descriptive and seldom integrate classical oxidation indices and fatty acid dynamics with metabolomic trajectories to resolve oxidation stages. In this work, hickory nut oil was subjected to accelerated thermal oxidation and sampled at predefined time points. Oxidative progression was evaluated by integrating physicochemical indices and fatty-acid profiling with untargeted LC–MS metabolomics, followed by multivariate modeling, differential-feature screening, and pathway enrichment to delineate stage-dependent oxidation dynamics. Despite its advantages, metabolomics-based studies on hickory nut oil remain limited. Integrating conventional analytical methods with metabolomics therefore offers a powerful approach for elucidating oxidative pathways and quality evolution in hickory nut oil, providing essential technical support for quality control and product development.

## 2. Materials and Methods

### 2.1. Materials and Reagents

Hickory nuts were purchased from the Changbai Mountain area in Jilin Province, China. Methanol, acetic acid, petroleum ether, boron trifluoride–methanol complex, acetonitrile, and isopropanol (all HPLC grade) were used for chromatographic analyses. All other chemicals were of analytical grade and used without further purification. These included hexane, toluene, sodium hydroxide, N-tert-butyl-α-phenylnitrone (PBN), chloroform, glacial acetic acid, potassium iodide, soluble starch, sodium thiosulfate, 1,1,3,3-tetraethoxypropane, trichloroacetic acid, thiobarbituric acid, benzene, 2,4-dinitrophenylhydrazine, potassium hydroxide, absolute ethanol and isooctane.

### 2.2. Experimental Methods

#### 2.2.1. Preparation of Hickory Oil

Cleaned kernels of hickory nuts were manually dehulled, peeled, and finely ground into a homogeneous powder. The resulting kernel meal was then mixed with n-hexane at a solid-to-solvent ratio of 1:5 (*w*/*v*) and subjected to static extraction for 12 h at room temperature under light-protected conditions. After extraction, the mixture was filtered, and the filtrate was concentrated using a rotary evaporator (RV 3 V, Schwerte, Germany) under reduced pressure until a constant weight was achieved, affording the final *C. cathayensis* oil sample.

#### 2.2.2. Accelerated Oxidation Experiment

Aliquots (10 mL each) of hickory nut oil were placed in beakers and subjected to accelerated thermal oxidation in a thermostatic oven at 60 °C [9]. Oxidation was performed with *n* = 3 process replicates (parallel vessels from the same oil batch). Throughout the 35-day experimental period, triplicate samples were collected at 5-day intervals, starting from day 0, for subsequent analysis of oxidative stability indices [10].

#### 2.2.3. Determination of Fatty Acid Composition

Sample pretreatment was performed following the method described by Mojsak et al. [11]. Fatty acid methyl esters were analyzed using a gas chromatography–mass spectrometry (GC–MS; Agilent 7890A-7000B, Santa Clara, CA, USA) system equipped with a capillary column (30 m × 250 μm × 0.25 μm; Agilent J&W Scientific, Folsom, CA, USA). Helium was used as the carrier gas at a constant flow rate of 1.0 mL/min. The injection volume was 1 μL with a split ratio of 10:1, and the injector temperature was maintained at 250 °C.

The oven temperature program was set as follows: initially 80 °C for 2 min, ramped to 140 °C at 30 °C/min and held for 1 min, then increased to 240 °C at 2 °C/min and maintained for 5 min. The GC–MS interface temperature was kept at 250 °C. Electron impact (EI) ionization was applied at 70 eV, and mass spectra were acquired in the *m*/*z* range of 40–450.

#### 2.2.4. Determination of Physicochemical Properties During the Oxidation of Hickory Nut Oil

POV was quantified by iodometric titration as described by Sabolová et al. [12]. The oil sample was dissolved in an acetic acid–chloroform mixture and subsequently reacted with excess potassium iodide to liberate iodine from hydroperoxides. The released iodine was then titrated with standardized sodium thiosulfate solution, employing starch as the endpoint indicator.

MDA content was analyzed according to Liu et al. [13]. Oil extracts were reacted with thiobarbituric acid (TBA) under acidic conditions at elevated temperature to generate a chromogenic adduct. The absorbance was subsequently measured spectrophotometrically against a calibration curve prepared from 1,1,3,3-tetraethoxypropane (TEP) standards.

COV was measured as described by Zamora et al. [14] based on the 2,4-dinitrophenylhydrazine (DNPH) derivatization of carbonyl compounds. Briefly, an oil aliquot was dissolved in an appropriate organic solvent and reacted with DNPH reagent under acidic conditions to form the corresponding hydrazones. After completion of derivatization, the reaction mixture was neutralized and/or diluted as required, and the hydrazone-containing phase was clarified (e.g., by brief centrifugation or filtration) prior to spectrophotometric measurement at the characteristic wavelength with a reagent blank processed in parallel.

CDV was assessed based on the procedure of Nguyen et al. [15] by UV absorbance measurement of conjugated dienes formed during lipid oxidation. Oil was diluted in an appropriate UV-transparent solvent (e.g., isooctane), and absorbance at 232 nm was recorded against a solvent blank.

### 2.3. Oxidative Metabolomics of Hickory Nut Oil

#### 2.3.1. Preparation of Hickory Nut Oil Samples at Various Oxidation Levels

Eight hickory nut oil samples were collected after accelerated oxidation at 60 °C for 0, 5, 10, 15, 20, 25, 30, and 35 days, and were designated as Y0d through Y35d, respectively. To ensure analytical consistency, a pooled quality control (QC) sample was prepared by combining equal aliquots from each individual sample. The resulting mixture was vortexed for 1 min to achieve homogeneity prior to vial transfer for subsequent analysis.

#### 2.3.2. UPLC–MS Analysis

Metabolomic profiling of hickory nut oil samples was performed using a Thermo Vanquish UPLC system coupled with a high-resolution mass spectrometer (both from Thermo Fisher Scientific, Carlsbad, CA, USA). Chromatographic separation was carried out on an ACQUITY UPLC^®^ HSS T3 column (2.1 × 100 mm, 1.8 μm; Waters, Milford, MA, USA) maintained at 40 °C, with a flow rate of 0.3 mL/min and an injection volume of 2 μL.

In positive ionization mode, the mobile phases consisted of water with 0.1% formic acid (A2) and acetonitrile with 0.1% formic acid (B2). In negative ionization mode, the mobile phases were 5 mM ammonium formate in water (A3) and acetonitrile (B3). Mass spectrometric detection was performed using an electrospray ionization (ESI) source in both positive and negative ion modes. The source parameters were set as follows: spray voltage, +3.50 kV (positive) and −2.50 kV (negative); capillary temperature, 325 °C; sheath gas flow rate, 40 arb; and auxiliary gas flow rate, 10 arb.

### 2.4. Statistical Analysis

All experimental analyses were performed in triplicate, and data are expressed as mean ± standard deviation. Statistical analysis was conducted using SPSS software (version 20.0), and data visualization was performed with Origin (version 2018). For metabolomic profiling, raw data were converted using the MSConvert tool (ProteoWizard software suite; version 3.0.22048) and subsequently processed with XCMS for peak picking and alignment. Metabolite identification was achieved by querying accurate mass and spectral data against public databases, including HMDB, LipidMaps, MassBank, mzCloud, and KEGG. Annotation followed standardized matching criteria. Precursor mass error was constrained to ≤5 ppm. MS/MS-based assignments required a spectral similarity score ≥ 0.80, concordant diagnostic fragment ions, and retention behavior consistent with the expected lipid-class elution order. Annotation confidence was classified according to the metabolomics standards initiative (MSI) criteria. The majority of lipid features were assigned as MSI Level 2 (putatively annotated compounds) based on MS/MS spectral matching to high-confidence reference libraries (e.g., LipidMaps, METLIN, and NIST). A targeted subset of key analytes—including predominant fatty acids and representative lipid oxidation products—was further verified as MSI Level 1 using authentic reference standards, supported by concordant retention times and MS/MS spectra. Features lacking definitive compound-level evidence but supported by accurate mass and class-diagnostic fragmentation patterns were reported as MSI Level 3 (putatively characterized compound classes), whereas features with insufficient structural information were retained as MSI Level 4 (unknowns). Univariate *p*-values were adjusted using the Benjamini–Hochberg false discovery rate (FDR) procedure; metabolites were defined as significantly changed only when meeting *p* < 0.05, FDR-adjusted *q* < 0.05, and OPLS-DA VIP > 1. Metabolic pathway enrichment analysis of differentially abundant metabolites was then performed based on the KEGG database (https://www.kegg.jp/, accessed on 18 March 2025).

## 3. Results

### 3.1. Changes in the Physicochemical Properties of Hickory Nut Oil During Oxidation

Lipid oxidation is a multi-stage process that generates a wide spectrum of primary and secondary reaction products, necessitating an integrated evaluation of oxidation markers together with fatty acid composition to accurately assess oxidative stability. Hickory nut oil, characterized by high levels of unsaturated fatty acids and endogenous antioxidants such as carotenoids and squalene, possesses an intrinsic capacity to modulate oxidative reactions and mitigate quality deterioration. Accordingly, this study aimed to elucidate the oxidative pattern and quality evolution of hickory nut oil during accelerated oxidation by monitoring POV, MDA, COV, CDV, and carotenoid content.

**Figure 1:**
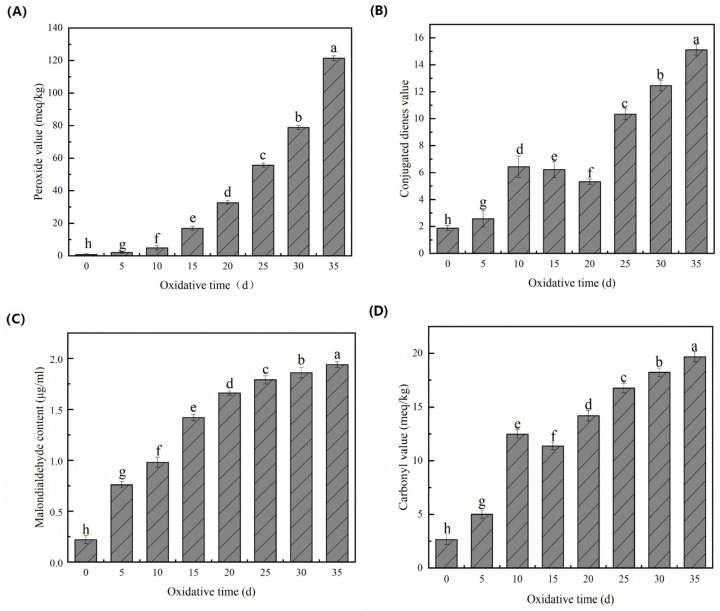
Variations in oxidative indices of hickory nut oil during the oxidation process. (**A**) Peroxide value (POV); (**B**) Conjugated diene value (CDV); (**C**) Malondialdehyde content (MDA); (**D**) Carbonyl value (COV). Different lowercase letters above the bars indicate significant differences among different oxidation times (*p* < 0.05).

The POV of hickory nut oil exhibited a significant increase throughout the oxidation process, with a markedly accelerated rate during the later stages (Figure 1A), indicating continuous accumulation of primary oxidation products. This trend is not unique to hickory nut oil but is consistently observed in other oils rich in polyunsaturated fatty acids (PUFAs). For instance, Alsufiani and Ashour [16] reported a progressive rise in the POV of sunflower oil during 88 days of ambient storage, which accelerated notably in the mid-to-late period. They attributed this pattern to the ongoing formation of hydroperoxides from unsaturated fatty acids, concurrent with the gradual depletion of endogenous antioxidants. Similarly, Kavran et al. [17] observed a persistent increase in the POV of sunflower oil under accelerated oxidation (40–80 °C), where the late-stage escalation was strongly influenced by temperature, initial POV, and the exhaustion of antioxidants. Furthermore, Sun et al. [18] documented the concomitant accumulation of POV and volatile aldehydes/ketones during walnut oil oxidation, suggesting that hydroperoxides accumulate continuously in the early phases before rapidly decomposing into secondary products. Therefore, under accelerated conditions, the unsaturated fatty acids in hickory nut oil continuously generate hydroperoxides. The depletion of endogenous antioxidants, together with intensified catalytic factors such as elevated temperature and metal ions, promotes both the accumulation and the subsequent accelerated decomposition of these hydroperoxides. This mechanistic cascade ultimately accounts for the pronounced late-stage surge in POV observed in this study.

The changes in the CDV of hickory nut oil during oxidation are shown (Figure 1B). The CDV exhibited an initial increase, followed by a transient decline after 15 days of accelerated oxidation, before rising markedly in later stages. This non-monotonic pattern—characterized by an initial rise, a mid-term drop, and a subsequent rebound—has been reported in various vegetable oils and lipid-rich foods and aligns with the behavior observed here in hickory nut oil. For example, Elsebaie et al. [19] noted a sharp CDV increase during early storage of cod liver oil-enriched meatballs, peaking on day 8 before declining. The authors attributed this decrease to the degradation of hydroperoxides and conjugated dienes into secondary oxidation products or their interaction with proteins, which reduces detectable conjugated double bonds. Similarly, A multi-laboratory study on rapeseed and sunflower oils also described an initial CDV increase, subsequent decrease, and final resurgence, linked to the exposure of new oxidation sites or accelerated oxidation dynamics [20]. In the present study, the early rapid CDV increase in hickory nut oil likely stems from the rearrangement of double bonds in unsaturated fatty acids into conjugated configurations, along with predominant hydroperoxide formation. The ensuing mid-stage decline corresponds to a shift where hydroperoxide decomposition surpasses formation, generating reactive secondary products such as aldehydes and ketones. These may undergo cross-linking or interact with other oil constituents, thereby reducing measurable conjugated dienes and CDV. As oxidation progresses, new reactive sites are exposed or oxidation intensifies, causing hydroperoxide and conjugated diene formation to again exceed decomposition—ultimately leading to the pronounced CDV rebound observed in the later phase.

The changes in MDA content in hickory nut oil during accelerated oxidation are shown (Figure 1C). The MDA level increased progressively throughout the oxidation process and stabilized after approximately 25 days. A similar trend was reported by Ma et al. [21] in a kinetic study of various vegetable oils, in which MDA content rose rapidly during the initial oxidation phase before reaching a plateau. The authors attributed this stabilization to a dynamic equilibrium between MDA formation and consumption, as the reactive aldehyde participates in further secondary reactions in later oxidation stages. Consistently, Domínguez et al. [22] demonstrated that the increase in MDA reflects the propagation phase of lipid oxidation in meat systems, characterized by accelerated free radical chain reactions. As oxidation progresses, the available pool of polyunsaturated fatty acids becomes progressively depleted, and free radicals recombine to form stable non-radical products, marking the onset of the termination phase. This leads to a deceleration and eventual stabilization of MDA formation. In the present study, the rapid accumulation of MDA during the early stage of oxidation in hickory nut oil is mainly due to peroxidation chain reactions of unsaturated fatty acids, where the rate of MDA generation substantially exceeds its consumption. As oxidation advances, the system transitions into a dynamic equilibrium governed by two concurrent mechanisms: substrate depletion and termination reactions. The gradual exhaustion of oxidizable unsaturated fatty acids reduces the rate of MDA formation, while radical recombination attenuates the propagation of lipid oxidation. Furthermore, MDA, being a highly reactive aldehyde, readily interacts with other oil constituents (e.g., proteins or amino acids) to form more complex and stable adducts, thereby enhancing its consumption. Together, these processes account for the stabilization of MDA levels in the later stages of oxidation.

The evolution of the COV in hickory nut oil during accelerated oxidation is shown (Figure 1D). The COV demonstrated a characteristic sigmoidal trend: it increased initially, underwent a transient decline around day 15, and then resumed a marked upward trajectory as oxidation progressed. This non-monotonic pattern aligns with behaviors reported in other edible oils. For instance, Ma et al. [23] observed that the COV in frying oils rose to a maximum before decreasing, a phenomenon they attributed to the decomposition of primary carbonyl compounds and the volatilization of low-molecular-weight derivatives. Similarly, Grebenteuch et al. [24] reported that during the accelerated oxidation of rapeseed oil, secondary carbonyls such as aldehydes were initially formed and later degraded, giving rise to more complex tertiary oxidation products and an increase in volatile organics. In the present study, the initial rapid rise in COV likely reflects the accumulation of primary carbonyl compounds derived from hydroperoxide decomposition. The subsequent decrease suggests a shift in reaction dynamics, where the rate of decomposition or conversion of these carbonyls exceeded their formation. As oxidation advanced, secondary reactions became dominant, leading to the formation of non-volatile, high-molecular-weight carbonyl species, which contributed to the observed rebound in COV. Moreover, in complex lipid systems like hickory nut oil, the breakdown of secondary products can regenerate carbonyl compounds, further driving the late-stage COV increase. Overall, the COV profile reflects a dynamic balance among the continuous generation, degradation, and transformation of carbonyl-containing compounds across different oxidation stages.

In summary, the oxidation of hickory nut oil progressed slowly during the initial phase, with a marked acceleration occurring only after 25 days. As oxidation advanced, a rapid increase in both POV and CDV indicated substantial hydroperoxide accumulation and ongoing fatty acid oxidation. Concurrently, the gradual rise in MDA content and COV reflected the steady formation of secondary oxidation products, such as aldehydes and related carbonyl species. Throughout this process, the buildup of peroxides and free radicals promoted the degradation of polyunsaturated fatty acids, generating off-flavor aldehydes and other volatile compounds that collectively contributed to the decline in oil quality. Notably, after 25 days, oxidation products accumulated rapidly, accompanied by a sharp deterioration in chemical quality, with potential sensory relevance inferred from the rapid accumulation of aldehydic oxidation products, further confirming the onset of significant oxidative degradation in hickory nut oil at this stage.

### 3.2. Fatty Acid Dynamics in Hickory Nut Oil During Oxidation

Six fatty acids were identified by gas chromatography (GC, 7890A, Agilent Technologies, CA, USA) in fresh hickory nut oil (day 0). These included palmitic acid (C16:0), stearic acid (C18:0), oleic acid (C18:1), linoleic acid (C18:2), linolenic acid (C18:3), and gadoleic acid (C20:1). The fatty acid profile was dominated by oleic, linoleic, and linolenic acids, which collectively accounted for over 90% of the total content, while the remaining three saturated and monounsaturated fatty acids constituted approximately 10%.

This study investigated the dynamic changes in the relative composition of major fatty acids in hickory nut oil during a 35-day accelerated oxidation period (Table 1). At day 0, the oil displayed a characteristic high-oleic profile, with unsaturated fatty acids (UFAs) accounting for 92.52% of total lipids, of which oleic acid (C18:1) constituted the predominant component (63.16%). As oxidation progressed, notable compositional shifts occurred, driven primarily by oxidative degradation of unsaturated double bonds, thereby altering the relative proportions of polyunsaturated fatty acids (PUFAs), monounsaturated fatty acids (MUFAs), and saturated fatty acids (SFAs). These changes were largely attributable to the decline in linoleic (C18:2) and linolenic (C18:3) acids and their differential oxidation rates. By day 35, the total UFA content decreased from 92.52% to 90.65%, with substantial variation in degradation rates among individual unsaturated species. Data revealed that PUFAs were the most labile, declining from 70.82% to 69.17%. Notably, linolenic acid (C18:3), with three double bonds, underwent the most pronounced reduction (from 7.66% to 7.41%, *p* < 0.05). This trend is closely linked to fatty acid molecular structure, as oxidation susceptibility increases with the number of double bonds. The methylene-interrupted bis-allylic hydrogens in PUFAs are particularly prone to hydrogen abstraction, initiating and propagating radical-mediated oxidation cascades. In line with this, Kazuo [25] emphasized that the high oxidizability of PUFAs stems from the presence of multiple double bonds, where bis-allylic hydrogen atoms readily undergo radical attack, thereby accelerating lipid oxidation.

Oleic acid (C18:1), as the predominant fatty acid in hickory nut oil, underwent a gradual yet statistically significant decrease from 63.16% to 61.76% (*p* < 0.05) during oxidation. Although MUFAs exhibit greater stability than their polyunsaturated counterparts, they remain susceptible to oxidative degradation under prolonged conditions. This observation is consistent with findings by El Bernoussi et al. [26], who reported a notable decline in oleic acid content in almond oil during accelerated storage, accompanied by a corresponding deterioration in overall oil quality. Their results further corroborate that MUFAs are not entirely resistant to extended oxidative stress.

Concurrent with the degradation of unsaturated fatty acids, the relative proportion of SFAs showed a significant increase from 6.92% to 7.88% (*p* < 0.05). This apparent rise is largely attributable to the oxidative loss of unsaturated fatty acids, which reduces their relative abundance and thereby elevates the proportion of chemically more stable SFAs. Consistently, Liang et al. [27] reported a similar relative increase in SFAs in thermally treated hemp seed oil, which they ascribed to the preferential oxidation of polyunsaturated fatty acids. Their findings offer a mechanistic explanation for the SFA trends observed in the present study.

### 3.3. Dual-Ionization Metabolomics Reveals Comprehensive Chemical Dynamics During Hickory Nut Oil Oxidation

**Figure 2:**
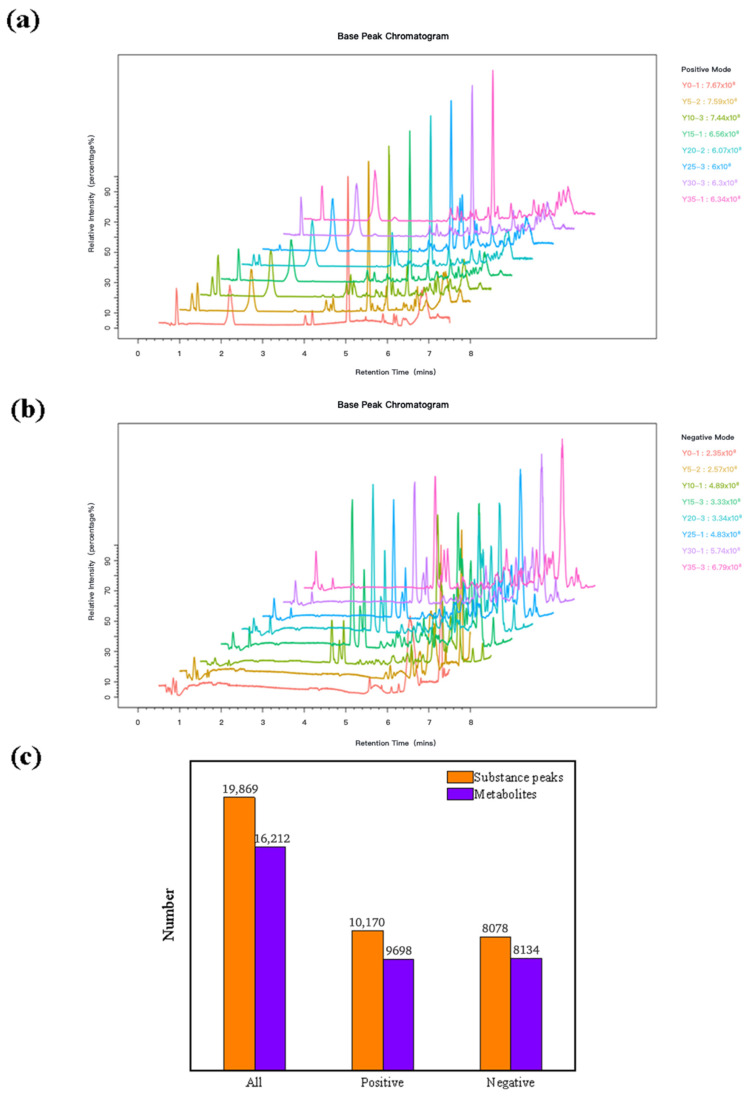
(**a**) Base peak chromatogram (BPC) of hickory nut oil in positive ion mode; (**b**) base peak chromatogram in negative ion mode; (**c**) statistical distribution of identified metabolites in hickory nut oil.

Samples were analyzed using a high-resolution mass spectrometer (Thermo Fisher Scientific, Carlsbad, CA, USA) equipped with an electrospray ionization (ESI) source operated in both positive and negative ionization modes. Given the wide range of physicochemical properties among metabolites, ionization efficiency and charge state vary substantially across different compounds. To ensure comprehensive metabolomic coverage, both positive (NT-pos) and negative (NT-neg) ionization modes were utilized. The base peak chromatograms (BPCs) of hickory nut oil at different oxidation stages under both ionization modes are shown (Figure 2a,b). Statistical processing of the LC–MS data identified a total of 19,869 ion features across the 35-day oxidation period (Figure 2c). Among these, a slightly higher number of features were detected in positive ion mode (10,170) compared to negative ion mode (8078). This disparity likely reflects differences in ionization efficiency, with the former being more favorable for compounds that readily undergo protonation—such as basic metabolites and other species with high proton affinity—under the employed chromatographic and mass spectrometric conditions.

Further metabolite annotation identified 16,212 lipid-related molecular features from the detected ion features. Consistent with the overall feature distribution, the positive ion mode contributed the majority of annotated metabolites. Interestingly, despite the lower total number of features in the negative ion mode (8078), the number of metabolites identified in this mode (8134) slightly exceeded the feature count. This discrepancy may arise from the annotation of multiple isomeric metabolites to a single chromatographic feature, or from overlapping metabolite assignments during data processing. Overall, the positive ion mode demonstrated broader coverage for lipid oxidation products and amino acid derivatives, while the combined use of both ionization modes proved essential for comprehensively elucidating the metabolic changes during the oxidation of hickory nut oil.

### 3.4. Unsupervised Multivariate Discrimination and Metabolomic Profiling of Hickory Nut Oil

**Figure 3:**
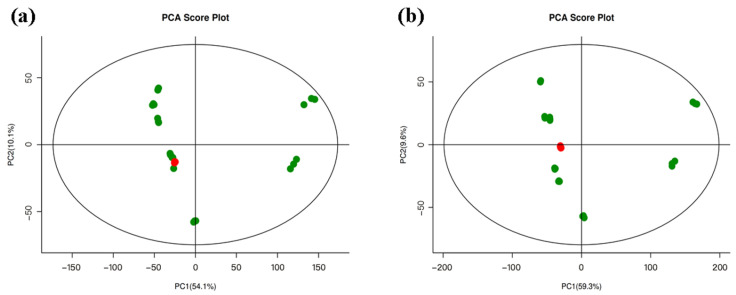
Principal component analysis (PCA) score plots of QC samples obtained under (**a**) positive (NT-pos) and (**b**) negative (NT-neg) ionization modes. The red dots indicate QC samples, whereas the green dots indicate experimental samples.

Principal component analysis (PCA) was performed to evaluate analytical stability and inter-sample metabolic differences (Figure 3). In both positive (NT-pos) and negative (NT-neg) ionization modes, the quality control (QC) samples (red dots) formed a tight cluster near the origin of the score plot, demonstrating high reproducibility and analytical stability throughout the sequence and thereby validating the reliability of the acquired data. In contrast, the experimental samples (green dots) exhibited clear clustering according to their intrinsic compositional profiles, reflecting systematic metabolic variation across oxidation stages. The first principal component (PC1) accounted for more than 54% of the total variance, indicating that sample differences were driven predominantly by progressive oxidation-related changes in the oil’s metabolome. Moreover, the consistent clustering patterns observed in both ionization modes provided cross-validation of the metabolic trends. Together, these PCA results confirm the robustness of the LC–MS platform and reveal significant metabolic divergence among samples, establishing a solid basis for subsequent biomarker discovery and mechanistic investigation.

**Figure 4:**
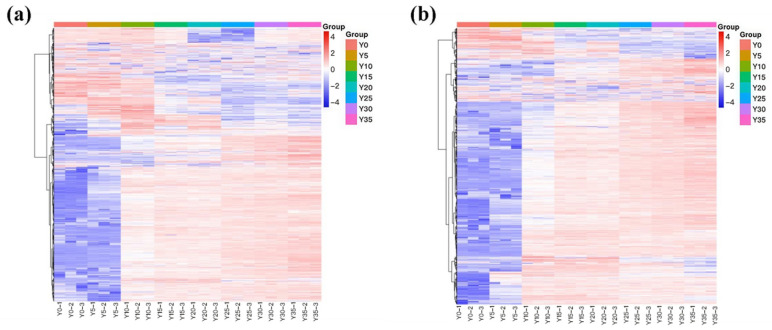
Hierarchical clustering heatmap of hickory nut oil samples under NT-pos (**a**) and NT-neg (**b**) ionization modes.

The metabolic profiles of hickory nut oil samples at varying oxidation levels were further investigated using hierarchical cluster analysis (HCA) to assess sample stability and inter-group correlations, including QC samples. Samples were clearly segregated into two major clusters—the early oxidation stage (Y0–Y5) and the accelerated oxidation stage (Y10–Y35) (Figure 4). A pronounced separation between Y5 and Y10 indicated a critical transition in the oil’s metabolic state. During the initial phase, changes were primarily associated with the gradual depletion of endogenous antioxidants and the accumulation of primary oxidation products such as hydroperoxides, consistent with the induction period of lipid oxidation. From Y10 onward, the heatmap revealed extensive alterations in metabolite abundance, signifying the onset of an accelerated oxidation phase dominated by the decomposition of primary products and the formation of various secondary oxidation compounds—including aldehydes and ketones—which collectively contribute to rapid quality deterioration.

### 3.5. Analysis of Differential Metabolites During Hickory Nut Oil Oxidation

**Figure 5:**
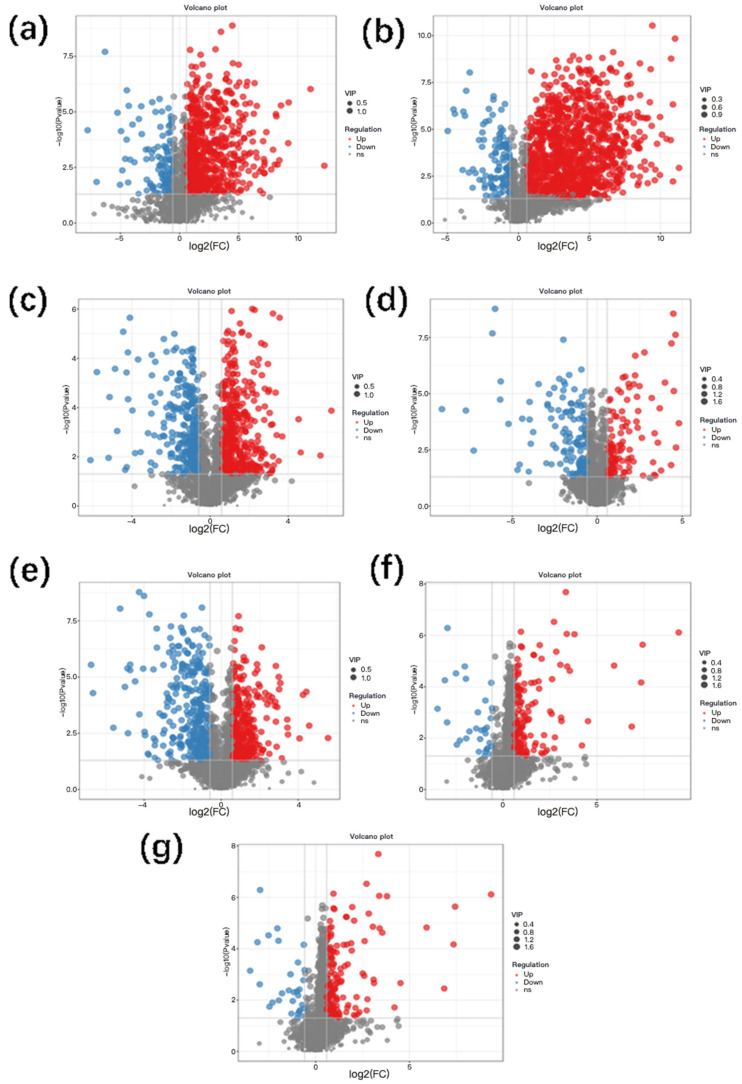
Volcano plots of differential metabolites in hickory nut oil across seven comparison groups: (**a**) Y5d vs. Y0d; (**b**) Y10d vs. Y5d; (**c**) Y15d vs. Y10d; (**d**) Y20d vs. Y15d; (**e**) Y25d vs. Y20d; (**f**) Y30d vs. Y25d; and (**g**) Y35d vs. Y30d. Blue, red, and gray dots represent downregulated, upregulated, and non-significant metabolites, respectively.

**Figure 6:**
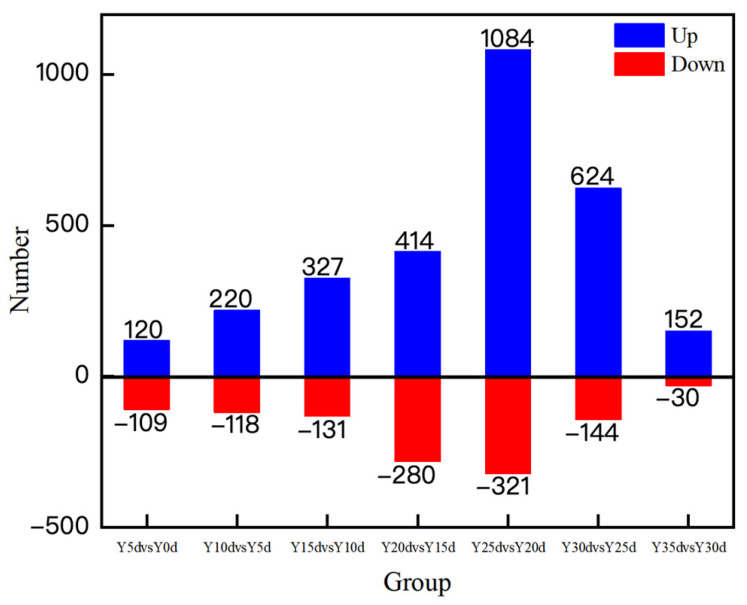
Statistical summary of differential metabolites derived from volcano plots across seven hickory nut oil comparisons.

To gain mechanistic insight into the oxidative deterioration process, we characterized the metabolic profiles of hickory nut oil across progressive oxidation stages to delineate the trajectories of its metabolic alterations. Differentially abundant metabolites were first screened using Student’s *t*-test combined with fold change (FC) analysis, followed by orthogonal partial least squares–discriminant analysis (OPLS-DA) to resolve systemic metabolic variations among sample groups. Model performance was acceptable, with cumulative R^2^Y and Q^2^ values of 0.94 and 0.82, respectively. Metabolites with variable importance in projection (VIP) values > 1 and *p* < 0.05 were considered statistically significant. Volcano plots and statistical summaries (Figure 5 and Figure 6) visualize the distribution and dynamic variation of these metabolites. Compared with the Y0d control, analysis identified 120, 220, 327, 414, 1084, 624, and 152 upregulated metabolites and 109, 118, 131, 280, 321, 144, and 30 downregulated metabolites in Y5d–Y35d samples, respectively. A markedly steeper increase in upregulated metabolites indicated that the formation rate of oxidative products outpaced the consumption of endogenous constituents as oxidation intensified. This pattern aligns with the canonical mechanism of lipid autoxidation, which progresses through initiation, propagation, and termination. In the early-to-intermediate stages (Y5d–Y25d), unsaturated fatty acids reacted with oxygen to yield unstable hydroperoxides, whose rapid decomposition initiated a cascade of chain reactions, culminating in an exponential accumulation of secondary oxidation products such as aldehydes, ketones, and carboxylic acids. As oxidation advanced beyond Y25d, extensive depletion of polyunsaturated fatty acids and endogenous antioxidants, together with enhanced radical recombination, drove the system toward the termination phase [28]. Consequently, the formation of new metabolites declined, and intermediates underwent polymerization or volatilization, collectively resulting in a reduced pool of detectable differential metabolites. Collectively, these findings pinpoint Y25d as a clear transition stage under accelerated oxidation marking the onset of oxidative deterioration in hickory nut oil, offering robust metabolomic evidence for elucidating its oxidation kinetics and stability thresholds.

Based on FC values, the top 50 differential metabolites were statistically analyzed to characterize compositional shifts across oxidation stages (Table 2). These metabolites included organophosphates and their derivatives, oxygen-containing organic compounds, isoprenoid lipids, phenylpropanoids, alkaloids, terpenoids, and other minor constituents. Across the seven comparison groups, the majority of the top 50 metabolites displayed an upward trend. Notably, carboxylic acids and their derivatives increased consistently with prolonged oxidation, representing a hallmark signature of lipid oxidation. As established by Spickett [29], hydroperoxides derived from unsaturated fatty acids undergo homolytic cleavage under thermal or metal-catalyzed conditions, generating highly reactive alkoxyl radicals. These radicals subsequently undergo β-scission to form short-chain aldehydes, which are rapidly oxidized into corresponding carboxylic acids due to the high reactivity of the aldehyde group. This leads to the accumulation of various short- and medium-chain fatty acids. Therefore, the progressive buildup of carboxylic acids not only serves as a direct molecular indicator of the advancing oxidation in hickory nut oil but also underlies the increase in acid value and the development of rancid, pungent off-flavors. The data clearly reflect this dynamic transition from primary oxidation products to secondary and terminal oxidation species.

Conversely, in contrast to the upregulation of carboxylic acids, benzene and its substituted derivatives exhibited a marked downregulation trend. Endogenous antioxidants in vegetable oils, many of which contain aromatic moieties (e.g., phenolic constituents), contribute to radical scavenging capacity; representative examples include tocopherols and polyphenols. In this study, tocopherols, total polyphenols, and carotenoids were not directly determined by dedicated targeted assays; therefore, their changes are discussed in the context of the observed metabolomic trends. As noted by Weinbrenner et al. [30], during the early and intermediate oxidation stages, these phenolic compounds donate hydrogen atoms to scavenge free radicals, effectively interrupting oxidation chain reactions while being progressively consumed or structurally modified. Thus, the observed decline in benzene-containing compounds provides direct evidence of the gradual depletion of endogenous antioxidant capacity within the oil matrix. This trend further implies that after 25 days of oxidation, the overall number of differential metabolites began to decline, signaling that both oxidation substrates (unsaturated fatty acids) and intrinsic antioxidants had been substantially depleted. At this stage, the oil lost its native protective barrier and entered an irreversible phase of rapid quality deterioration.

Overall, the metabolic characteristics identified in the terminal oxidation stage provide critical theoretical insights into the shelf-life limits and quality deterioration mechanisms of hickory nut oil. Collectively, our findings delineate the oxidative trajectory from initiation to termination, elucidating the molecular events that underpin flavor deterioration and physicochemical instability during prolonged storage or processing. This study thereby deepens the mechanistic understanding of oxidative degradation in nut oils and establishes a critical theoretical foundation for developing strategies to enhance their oxidative stability and preserve sensory quality.

### 3.6. Screening of Differential Intermediate Metabolites in Hickory Nut Oil During Oxidation

Analysis of FC values in differential metabolites over the 35-day oxidation of hickory nut oil identified seven predominant compound classes (Table 3), including acyl lipids, carboxylic acids and their derivatives, and benzenes. These systematic compositional shifts clearly trace the chain-reaction mechanism characteristic of lipid autoxidation.

The temporal evolution of key metabolites revealed a triphasic trajectory of oxidative deterioration. In the initial phase (Y0–Y10), unsaturated fatty acids were rapidly oxidized to form unstable hydroperoxides, whose decomposition drove a marked upregulation of downstream metabolites such as benzenes, carboxylic acids, and fatty acyls. These low-molecular-weight species—including aldehydes, ketones, and alcohols—include aroma-relevant oxidation products widely associated with early rancid off-flavors, signaling the onset of quality deterioration [31]. During the intermediate phase (Y10–Y25), hydroperoxides underwent extensive breakdown, and secondary oxidation products such as aldehydes and ketones were further transformed. Metabolically, this stage was characterized by the gradual enrichment of isoprenoid lipids and phenolic compounds, alongside a decline in reactive oxygenated intermediates, reflecting a progressive intensification of oxidative rancidity. In the terminal phase (Y25–Y35), continued oxidation of aldehydes and ketones led to a pronounced accumulation of carboxylic acids and their derivatives, directly contributing to the sharp rise in acid value. Concurrently, the marked depletion of benzenes likely resulted from their participation in polymerization reactions, forming high-molecular-weight aggregates that promote color darkening, increased viscosity, and severe quality loss [32]. Additionally, the sustained upregulation of organophosphate esters may be associated with oxidative degradation of endogenous phospholipids.

### 3.7. Pathway Enrichment Profiling of Hickory Nut Oil Across Oxidation Stages

Pathway enrichment analysis was conducted to identify significantly altered metabolic pathways in hickory nut oil during oxidation. A pathway was considered statistically significant at *p* < 0.05, with smaller *p*-values indicating higher significance. The results were visualized using a bubble plot, where the x-axis depicts the pathway impact value and the y-axis shows pathway names. In this representation, node color intensity (blue to red gradient) corresponds to the *p*-value—redder hues denote lower *p*-values and greater significance—while node size reflects the number of enriched metabolites per pathway. Accordingly, KEGG enrichment results are interpreted here as an annotation-driven, exploratory (hypothesis-generating) framework for organizing oxidation-derived features, recognizing that pathway labels—including disease- or endocrine-related terms and human/animal-labeled maps—primarily arise from structural similarity and compound-set overlap with KEGG-curated metabolites, rather than indicating biological process activation or physiological regulation in this non-living matrix.

**Figure 7:**
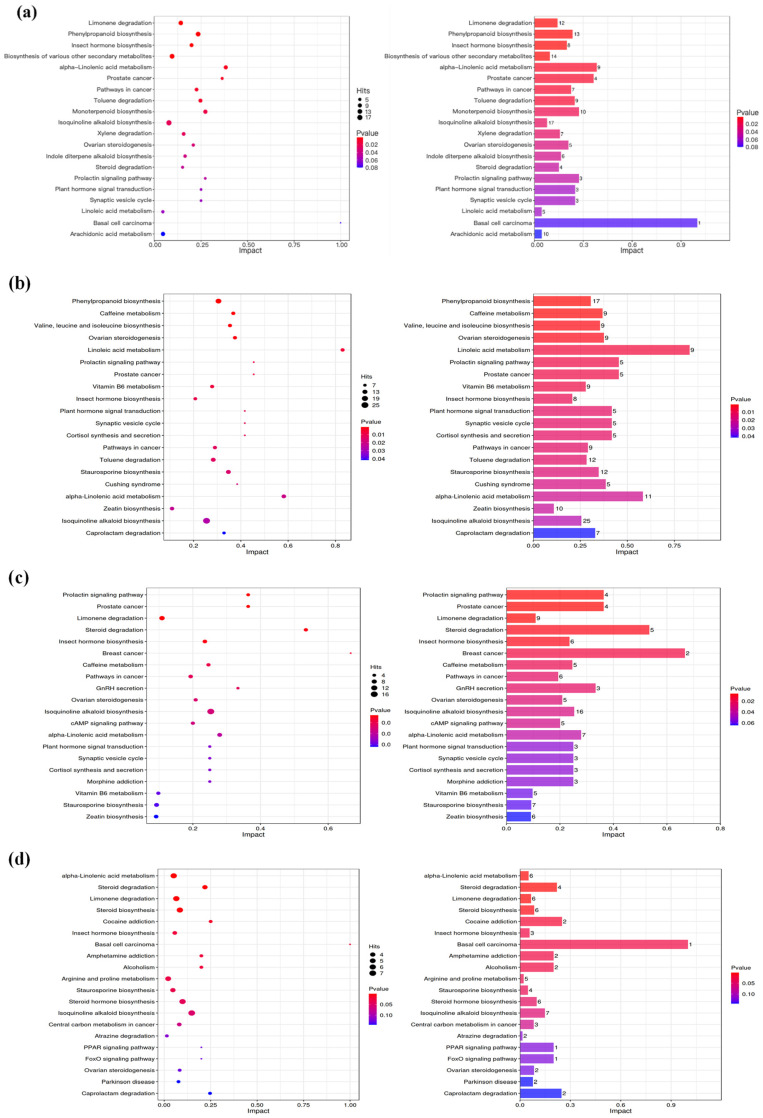

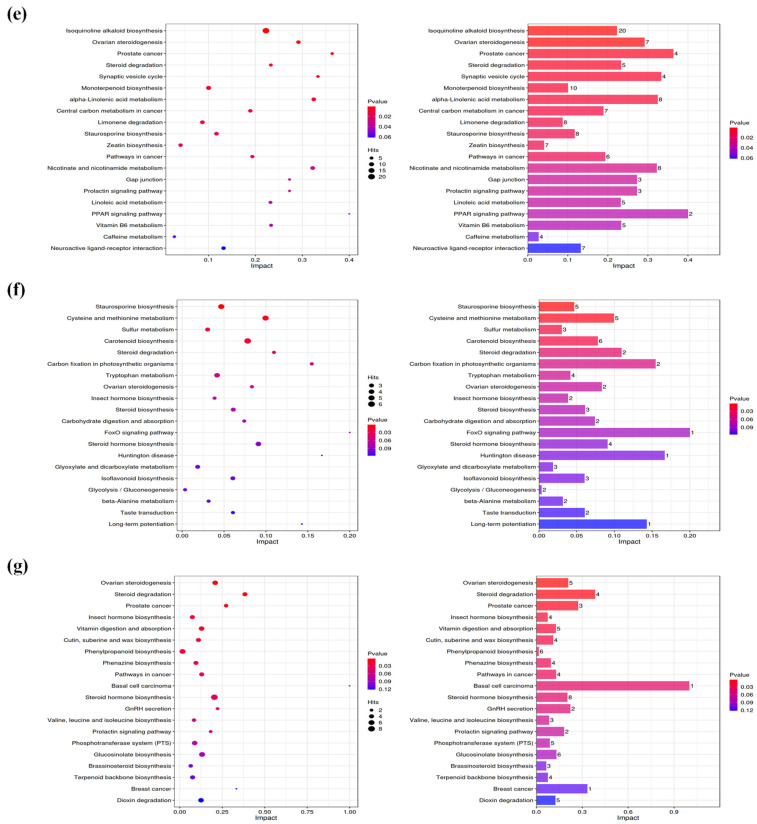
Bubble plot and bar chart of metabolic pathway impact factors; (**a**) Y5d vs. Y0d; (**b**) Y10d vs. Y5d; (**c**) Y15d vs. Y10d; (**d**) Y20d vs. Y15d; (**e**) Y25d vs. Y20d; (**f**) Y30d vs. Y25d; (**g**) Y35d vs. Y30d.

Pathway enrichment analysis of hickory nut oil following 5 days of accelerated oxidation revealed significant enrichment of differential metabolites in multiple metabolic pathways, as visualized in the corresponding bubble and bar plots (Figure 7a). The most prominently enriched pathways included α-linolenic acid metabolism, linoleic acid metabolism, and arachidonic acid metabolism, indicating that UFAs—the core lipid substrates—were undergoing vigorous free-radical chain oxidation. This process led to the generation of hydroperoxides, a hallmark of early-stage lipid oxidation. Concurrently, several biosynthetic pathways of secondary metabolites—such as phenylpropanoids, monoterpenoids, and isoquinoline alkaloids—were also significantly enriched, reflecting changes in antioxidant-related constituents and their associated metabolites during chemical oxidation. Notably, the phenylpropanoid pathway plays a pivotal role in the synthesis of phenolic compounds, including phenolic acids and flavonoids. These endogenous antioxidants help mitigate oxidative damage by quenching free radicals via hydrogen atom transfer, thereby interrupting UFA oxidation chain reactions and fulfilling a central regulatory function [33].

Thus, the metabolic network during initial oxidation reflects a dynamic competition between the oxidative degradation of PUFAs and the counteractive regulation by endogenous secondary metabolites. This fundamental interplay between lipid peroxidation and plant secondary metabolism critically shapes the trajectory and kinetics of the early oxidation process.

After 10 days of oxidation, analysis of the significantly enriched metabolic pathways (Figure 7b) indicated a clear transition in the lipid oxidation profile. Linoleic acid metabolism showed the highest impact factor, marking a shift in the oxidative mechanism from initial hydroperoxide accumulation to widespread formation of secondary products such as aldehydes and ketones. The strong enrichment of phenylpropanoid biosynthesis highlights the accelerated consumption of endogenous phenolic antioxidants under persistent oxidative stress. Concurrent activation of branched-chain amino acid metabolism (valine, leucine, and isoleucine) further reveals the extension of oxidative damage to proteins and free amino acids. During this stage, abundant reactive aldehydes can covalently modify amino acids through pathways like Strecker degradation, resulting in novel derivatives. In summary, the metabolic profile at this stage extends beyond lipid peroxidation, reflecting the onset of a multifaceted deterioration cascade—including depletion of antioxidant constituents and intensified secondary-product formation—indicating a transition from antioxidant depletion to accelerated chemical degradation and structural breakdown of oil constituents.

After 15 days of oxidation, analysis of the core enriched pathways (Figure 7c) identified steroid degradation, isoquinoline alkaloid biosynthesis, cAMP signaling, and α-linolenic acid metabolism as the most significantly enriched (*p* ≤ 0.02). Steroid degradation exhibited both the highest enrichment and impact factor, suggesting activation of a degradation-associated transformation to eliminate structurally compromised steroid molecules, thereby mitigating potential cytotoxicity and potentially mitigating the accumulation of oxidation-derived reactive species. Intracellularly, marked activation of the cAMP signaling pathway was annotated as significantly perturbed, consistent with broad shifts in the metabolite annotation network during oxidation. This was accompanied by mobilization of defense-related biosynthesis: α-linolenic acid metabolism promoted the formation of oxidized lipids with anti-inflammatory and pro-resolving properties, while isoquinoline alkaloid biosynthesis and limonene degradation indicated direct antioxidative activity. Together, these responses reflect a metabolically adaptive network, with steroid degradation likely serving a central regulatory role in this remodeling process.

After 20 days of oxidation, pathway enrichment analysis (Figure 7d) highlighted α-linolenic acid metabolism, steroid degradation, limonene degradation, and arginine/proline metabolism as the most affected (*p* ≤ 0.05). The pronounced enrichment of α-linolenic acid metabolism reaffirmed polyunsaturated fatty acids as primary oxidation substrates and indicated progression from hydroperoxide buildup to extensive secondary carbonyl formation—marking an accelerated autocatalytic phase. Co-enrichment of steroid and limonene degradation revealed oxidative damage extending to lipid-soluble minor components, including phytosterols and terpenoid flavor compounds, resulting in concomitant loss of nutritional and sensory quality. Although the basal cell carcinoma pathway lacked high statistical significance (*p* > 0.05), its top impact factor suggests a topologically pivotal role in the metabolic network. Together with isoquinoline alkaloid biosynthesis, these findings illustrate the complexity and pleiotropy of late-stage metabolic regulation.

After 25 days of oxidation, metabolic profiling (Figure 7e) showed significant enrichment of isoquinoline alkaloid biosynthesis and ovarian steroidogenesis (*p* ≤ 0.02), with the former containing substantially more metabolites (Hits = 20) than other pathways. This indicates that oxidation was associated with marked compositional shifts in the residual phytochemical matrix, extending damage beyond primary lipid oxidation. Systemic activation of hormone- and steroid-related pathways—such as prostate cancer signaling and steroid degradation—was also evident. The prostate cancer pathway displayed the highest impact factor, reflecting severe disruption of central signaling hubs. Pathways related to persistent lipid peroxidation (e.g., α-linolenic acid metabolism) and plant secondary metabolism (e.g., monoterpenoid biosynthesis) remained significantly perturbed. Products of α-linolenic acid metabolism may act as endogenous signaling molecules that interfere with steroid hormone synthesis and homeostasis. Thus, this stage is defined by a shift from lipid-centric deterioration to oxidation-driven compositional remodeling, signaling a transition from disrupted signaling to incipient functional consequences at the cellular level.

Metabolic profiling after 30 days of oxidation (Figure 7f) indicated a transition from oxidative damage to a phase showing stronger enrichment of antioxidant-related annotations. Cysteine/methionine metabolism and carotenoid biosynthesis emerged as the most significantly enriched pathways (*p* ≤ 0.03). The enrichment of cysteine/methionine metabolism reflects an increased supply of sulfur-containing precursors for glutathione synthesis, thereby inhibiting lipid peroxidation chain propagation. Simultaneously, enhanced carotenoid biosynthesis establishes a lipophilic antioxidant barrier that facilitates direct radical quenching in hydrophobic compartments. The marked enrichment of clavicipitic acid biosynthesis and sulfur metabolism further underscores the importance of sulfur-mediated mechanisms in late-stage antioxidant defense. Notably, the FoxO signaling pathway, although not the most statistically significant, exhibited the highest impact factor, emphasizing its central role in regulating stress response, apoptosis, and longevity. Collectively, this oxidative phase is defined by an integrated stress-resistance network that coordinates signal transduction, precursor availability, radical scavenging, and damage containment—characteristic of an adaptive metabolic reprogramming under sustained oxidation.

At the final oxidation stage (35 days), the metabolic profile (Figure 7g) identified ovarian steroidogenesis and steroid degradation as the most significantly enriched pathways, with impact factors substantially exceeding those of other pathways—reflecting extensive oxidation and structural breakdown of core phytosterols. Significant enrichment was also observed in phenylpropanoid biosynthesis and insect hormone biosynthesis. The metabolic signature at this terminal phase reveals a dual dynamic: activation of phenylpropanoid biosynthesis signifies persistent engagement of endogenous antioxidant mechanisms, whereas the high metabolite involvement in steroid hormone biosynthesis indicates that these defenses are insufficient to counteract extensive chemical degradation of key lipid constituents and loss of oxidative stability. Notably, signaling pathways such as basal cell carcinoma, though involving fewer metabolites, displayed anomalously high impact factors, suggesting that late-stage oxidation products (e.g., 4-hydroxy-nonenal) may disrupt critical signaling hubs—without implying actual carcinogenesis in this plant oil system. In summary, the endpoint of oxidation is defined by the failure of endogenous protection, structural disintegration of key lipids, and dysregulation of signaling networks, collectively leading to irreversible quality deterioration.

The chromatic evolution of hickory nut oil during the 35-day oxidation period is primarily driven by the progressive accumulation of lipid oxidation products and the concomitant formation of secondary metabolites, including phenolic compounds and pigment precursors. The continuous generation of reactive carbonyl species—particularly aldehyde-ketone peroxides—induces cleavage, adduction, and polymerization of endogenous pigments such as carotenoids and lutein, resulting in pronounced darkening and browning. Concurrently, phenylpropanoids and phenolic compounds, which constitute a key endogenous antioxidant system, are progressively consumed under sustained oxidative stress. This depletion coincides with the formation of new pigment precursors and a range of reactive intermediates that collectively exacerbate color deterioration. This mechanistic interplay is supported by Palanisamy et al. [34], who demonstrated that specific phenolic compounds significantly modulate color stability in stored fish muscle through complex interactions with lipid oxidation products and pigments, underscoring the synergistic role of secondary metabolism in accelerating chromatic degradation. Therefore, the observed color transitions represent not only the direct outcome of chemical reactions between lipids and pigments but also serve as a visible indicator of oxidation-driven compositional remodeling.

The oxidation process of hickory nut oil follows a non-linear chemical oxidation progression, divided into three sequential stages that evolve from initial substrate depletion to systemic dysregulation. During the early phase (0–5 days), PUFAs such as α-linolenic acid and linoleic acid undergo radical-initiated oxidation, while phenylpropanoid and monoterpenoid biosynthesis pathways are activated—reflecting a coexistence of direct lipid peroxidation and early stress response mechanisms. In the intermediate phase (5–25 days), oxidation extends to more complex lipid structures, including steroids. Enrichment of steroid degradation, ovarian steroidogenesis, and amino acid metabolism pathways indicates disruption of hormonal homeostasis and signaling networks, whereas reactive carbonyls derived from Strecker degradation further aggravate metabolic imbalance. In the late phase (25–35 days), a broad cytoprotective network is mobilized system-wide. However, this defense is counteracted by progressive structural disintegration of core lipids such as phytosterols. Deep oxidation products—including 4-hydroxy-nonenal—severely interfere with cellular signaling modules, as indicated by the aberrantly high impact factors of pathways associated with cellular pathophysiology (e.g., basal cell carcinoma), ultimately overwhelming the system’s compensatory capacity and leading to irreversible deterioration of oil quality.

This study systematically investigated the oxidation progression of hickory nut oil using a metabolomics approach, establishing an evidence-based staging system grounded in omics data. Under accelerated conditions (60 °C), three distinct oxidative phases were identified: early (0–5 days), intermediate (5–25 days), and late (beyond 25 days). Enrichment analysis of metabolic pathways revealed a clear transition from initial lipid peroxidation—involving α-linolenic acid and steroid metabolism—and activation of secondary defense pathways (e.g., phenylpropanoid and terpenoid biosynthesis) in the early stage, to pathways indicating oxidative damage extending to regulatory signaling and amino acid metabolism in the intermediate stage. During the late stage, a systemic stress response emerged, characterized by antioxidant precursor synthesis (e.g., cysteine and methionine metabolism) alongside extensive functional dysregulation. These findings illustrate that lipid oxidation is not merely a cascade of chemical degradation, but a system-level process involving network-wide reprogramming. The collapse of endogenous antioxidant defenses and disruption of key signaling pathways collectively mark the critical transition toward irreversible quality deterioration. Furthermore, highly reactive lipid oxidation products may integrate into metabolic regulation, thereby driving the transition to irreversible quality loss through both sensory and functional decline.

## 4. Discussion

Understanding the dynamic physicochemical evolution of oils under accelerated oxidation is critical for elucidating the mechanistic basis of lipid stability. In this study, the temporal evolution of physicochemical parameters, fatty acid composition, and metabolomic profiles of hickory nut oil was systematically characterized during a 35-day accelerated oxidation at 60 °C. The oxidative stability of hickory nut oil is predominantly governed by the interplay between reactive lipid substrates and endogenous antioxidant defenses. Collectively, these results demonstrate that lipid oxidation follows a non-linear, multi-phase kinetic behavior, rather than a simple linear progression. Mechanistically, within the Schaal-oven framework, such non-linearity is expected because the apparent trajectory of each index reflects a shifting balance between (i) initiation and propagation reactions that generate hydroperoxides and (ii) decomposition and secondary reactions that consume unstable intermediates and yield carbonyls, acids, and polymerization products. Accordingly, the timing of inflection points provides a practical, mechanism-informed descriptor of oxidative stability beyond end-point comparisons.

During the early oxidation phase (0–10 days), primary oxidation indices, including peroxide value and malondialdehyde content, increased progressively, reflecting the formation and accumulation of hydroperoxides and the early emergence of secondary aldehyde products. In contrast, the conjugated diene value and carbonyl value exhibited a non-monotonic temporal pattern, characterized by an initial maximum around day 15, followed by a transient decline and subsequent rebound. Similar behavior has been reported in walnut and sunflower oils and is indicative of a dynamic kinetic balance during the transition from the propagation to the termination stage of oxidation, wherein the decomposition of unstable hydroperoxides into secondary volatile products temporarily outpaces their formation [16,18]. These indices therefore reflect a net balance between formation and decomposition processes. The resurgence of carbonyl value during the late oxidation stage likely reflects the net accumulation of relatively stable carbonyl species together with concomitant polymerization/condensation products, which together mark an irreversible deterioration of oil quality. Importantly, when these patterns are interpreted alongside the full time-course dataset, a pronounced acceleration becomes evident after approximately Day 25. This stage is characterized by the continued rise in secondary oxidation indices (e.g., the sharper increase in COV) and the concurrent emergence of oxidation-derived LC–MS features, consistent with a transition from a partially buffered propagation regime to a secondary-product-dominated deterioration phase—an interpretation that aligns with the canonical sequence proposed for edible oils while providing a time-resolved boundary specific to hickory nut oil under 60 °C stress.

The progressive loss of physicochemical quality is intrinsically coupled to degradation of the lipid matrix. Fatty acid profiling revealed a sustained depletion of unsaturated fatty acids, particularly polyunsaturated fatty acids, during the intermediate oxidation period. Although monounsaturated fatty acids such as oleic acid are generally regarded as comparatively resistant to oxidation, a modest relative variability was observed, while saturated fatty acids appeared proportionally enriched as a consequence of unsaturated fatty acid consumption. The pronounced depletion of linolenic (C18:3) and linoleic (C18:2) acids can be mechanistically attributed to the presence of bis-allylic methylene groups, whose low C–H bond dissociation energies render them highly susceptible to radical-mediated hydrogen abstraction. Thermal stability analyses further suggest that these compositional shifts are driven primarily by the preferential oxidation of linoleic and α-linolenic acids and their distinct kinetic behaviors. This selective degradation aligns with the “unsaturation rule” of lipid oxidation kinetics, consistent with observations in almond oil [26] and other nut oils [23], supporting the view that the polyunsaturated-to-saturated fatty acid ratio is a key determinant of hickory nut oil shelf stability. However, fatty-acid percentage shifts may underestimate functional quality loss once chain propagation is established, as limited PUFA consumption can generate disproportionately large pools of reactive aldehydes and carbonyls that drive sensory deterioration. Integrating fatty-acid dynamics with carbonyl indices thus strengthens mechanistic coherence and enables meaningful cross-study comparison.

Beyond conventional lipid chemical indices, untargeted metabolomic analysis afforded molecular-level insight into the oxidative trajectory of hickory nut oil. Throughout the 35-day accelerated oxidation period, metabolic perturbations were predominantly associated with α-linolenic acid, linoleic acid, arachidonic acid, and their downstream oxidation products, as reflected by the high impact values of these pathways in enrichment analyses. Importantly, the lipid oxidation cascade was accompanied by marked fluctuations in secondary metabolites, including phenylpropanoids, monoterpenoids, and isoquinoline alkaloids. Early enrichment of phenylpropanoid biosynthesis (0–10 days) suggests the active mobilization of endogenous antioxidant systems, such as tocopherols and phenolic acids, as an adaptive response to oxidative stress. However, progressive depletion of these bioactive compounds ultimately culminated in a loss of metabolic homeostasis. Compared with many existing LC–MS oxidation studies that emphasize either volatile fingerprints or selected lipid subclasses, our study explicitly links time-resolved metabolomic features to synchronous changes in (i) established oxidation indices (POV/MDA/CDV/COV) and (ii) fatty-acid substrate depletion. This cross-validation across orthogonal evidence streams reduces reliance on any single marker class and provides a more reproducible basis for defining oxidation stages in a relatively under-studied nut oil matrix. In mechanistic terms, the observed metabolite trajectories are consistent with a classical radical-chain lipid autoxidation process initiated by hydrogen abstraction and followed by a stage-dependent shift from primary oxidation to secondary decomposition pathways. This kinetic progression can help rationalize the non-linear behavior of conventional indices (e.g., the mid-stage inflection of CDV/COV) together with the continued emergence of downstream oxidation-related metabolites captured by LC–MS. Notably, the sustained increase in organic phosphoric acid derivatives (organophosphate esters) suggests that, beyond triacylglycerol peroxidation, oxidative modification and/or degradation of endogenous phospholipids may contribute additional oxidation routes during advanced stages. These mechanistic interpretations are provided to contextualize the metabolomic trends and warrant further validation by targeted assays of key lipid oxidation intermediates and phospholipid subclasses.

Integration of metabolomic alterations with physicochemical indices and fatty acid compositional changes enabled the oxidation process to be delineated into three discrete stages: an early phase (0–5 days) dominated by hydroperoxide formation and antioxidant defense; an intermediate phase (5–25 days) characterized by secondary oxidation product accumulation and metabolomic profile perturbation; and a late phase (beyond 25 days) marked by extensive lipid structural breakdown and systemic oxidative failure. This stage-resolved framework provides a more mechanistically informative interpretation of lipid oxidation than conventional kinetic descriptions and underscores Day 25 as a critical tipping point governing the oxidative stability of hickory nut oil. Future studies may further integrate targeted quantification of representative oxidation-derived metabolites with kinetic modeling approaches to validate and refine the proposed stage-resolved framework, thereby enabling more precise identification of critical control points in lipid oxidation.

## 5. Conclusions

This study presents a comprehensive multi-omics characterization of the oxidative stability of hickory nut oil. By coupling traditional physicochemical assays with high-throughput metabolomics, the trajectory of lipid deterioration under accelerated conditions was systematically delineated. The oxidation process was primarily driven by the selective degradation of α-linolenic and linoleic acids, which act as key substrates in radical chain reactions. A triphasic oxidative progression was identified, comprising an early phase (0–5 days), an intermediate phase (5–25 days), and a late phase (beyond 25 days). The transition from antioxidant defense, involving phenylpropanoids and terpenoids, to widespread lipid metabolic dysregulation reflects the gradual loss of oxidative stability and the onset of rancidity. Overall, these findings elucidate the stage-dependent metabolic changes in hickory nut oil during accelerated oxidation and provide a valuable reference for further understanding its oxidation mechanisms and quality deterioration.

## Figures and Tables

**Table 1:** Changes in fatty acid composition of hickory oil at different stages of oxidation for 35 days.

Fatty Acids/(%)	Oxidation Time (d)							
	0	5	10	15	20	25	30	35
C16:0	3.94 ± 0.01 ^h^	3.98 ± 0.02 ^g^	4.02 ± 0.04 ^f^	4.05 ± 0.02 ^e^	4.08 ± 0.01 ^d^	4.11 ± 0.02 ^c^	4.14 ± 0.02 ^b^	4.16 ± 0.02 ^a^
C18:0	2.98 ± 0.01 ^e^	3.21 ± 0.01 ^d^	3.62 ± 0.02 ^c^	3.68 ± 0.01 ^b^	3.72 ± 0.02 ^a^	3.73 ± 0.01 ^a^	3.72 ± 0.01 ^a^	3.72 ± 0.01 ^a^
C18:1	63.16 ± 0.08 ^a^	63.12 ± 0.03 ^c^	63.14 ± 0.06 ^b^	62.88 ± 0.08 ^d^	62.74 ± 0.04 ^e^	62.31 ± 0.05 ^f^	61.97 ± 0.04 ^g^	61.76 ± 0.05 ^h^
C18:2	21.34 ± 0.04 ^e^	21.32 ± 0.01 ^d^	21.28 ± 0.05 ^c^	21.22 ± 0.04 ^b^	21.21 ± 0.07 ^ab^	21.22 ± 0.04 ^ab^	21.20 ± 0.03 ^a^	21.20 ± 0.06 ^a^
C18:3	7.66 ± 0.03 ^a^	7.62 ± 0.01 ^b^	7.59 ± 0.02 ^c^	7.54 ± 0.01 ^d^	7.51 ± 0.02 ^e^	7.47 ± 0.01 ^f^	7.43 ± 0.03 ^g^	7.41 ± 0.02 ^h^
C20:1	0.36 ± 0.01 ^a^	0.35 ± 0.02 ^a^	0.34 ± 0.01 ^a^	0.33 ± 0.01 ^a^	0.32 ± 0.02 ^a^	0.30 ± 0.01 ^a^	0.28 ± 0.01 ^a^	0.28 ± 0.01 ^a^
SFA	6.92 ± 0.02 ^c^	7.19 ± 0.03 ^b^	7.64 ± 0.06 ^a^	7.73 ± 0.03 ^a^	7.80 ± 0.03 ^a^	7.84 ± 0.03 ^a^	7.86 ± 0.03 ^a^	7.88 ± 0.03 ^a^
UFA	92.52 ± 0.16	92.41 ± 0.07	92.35 ± 0.14	91.97 ± 0.14	91.78 ± 0.15	91.30 ± 0.11	90.88 ± 0.11	90.65 ± 0.14
MUFA	21.7 ± 0.05	21.67 ± 0.03	21.62 ± 0.06	21.55 ± 0.05	21.53 ± 0.09	21.52 ± 0.05	21.48 ± 0.04	21.48 ± 0.07
PUFA	70.82 ± 0.11	70.74 ± 0.04	70.73 ± 0.08	70.42 ± 0.09	70.25 ± 0.06	69.78 ± 0.06	69.40 ± 0.07	69.17 ± 0.07

Note: Values are expressed as mean ± SD (*n* = 3), and superscripted lowercase letters in the peer group indicate significant differences (*p* < 0.05).

**Table 2:** Top 50 discriminative metabolites reflecting category-specific variation among hickory nut oil samples.

Metabolite Type		Y5d vs. Y0d	Y10d vs. Y5d	Y15d vs. Y10d	Y20d vs. Y15d	Y25d vs. Y20d	Y30d vs. Y25d	Y35d vs. Y30d
Fatty acyl	Up	5	7	4	7	4	7	5
	Down	1	-	3	2	3	1	2
Benzene and its derivatives	Up	8	11	6	6	3	4	4
	Down	4	1	4	1	5	2	4
Organic compound	Up	5	5	-	4	-	4	1
	Down	-	1	2	-	5	-	2
Carboxylic acid and its derivatives	Up	3	4	4	5	9	9	7
	Down	7	2	5	2	2	1	1
Isopentenol lipids	Up	1	1	3	-	-	-	-
	Down	-	-	1	3	-	-	-
Organic nitrogen compound	Up	3	2	-	3	-	-	2
	Down	-	2	1	-	2	-	-
Phenylpropanoid	Up	2	1	2	1	1	1	1
	Down	-	-	-	3	2	1	-
Keto acids and their derivatives	Up	-	1	-	-	-	-	-
	Down	-	-	1	-	-	-	-
Else	Up	9	10	8	7	2	11	6
	Down	1	-	5	3	10	4	8
Alkaloid	Up	1	-	-	1	1	-	-
	Down	-	-	1	2	-	-	-
Organic phosphoric acid and its derivatives	Up	-	2	-	-	-	2	4
	Down	-	-	-	-	-	1	1
Terpenoid	Up	-	-	-	-	-	2	-
	Down	-	-	-	-	1	-	1

**Table 3:** Representative intermediate metabolites characterizing the oxidative transition in hickory nut oil.

Group	Metabolite	Classification	FC	Type
Y5 vs. Y0	2-Hydroxybenzyl alcohol	Benzene and its derivatives	12.59	Up
	Diethanolamine	Organic Nitrides	3.57	Up
	3-Chlorobenzoic acid	Benzene and its derivatives	2.49	Up
	L-Serine	Carboxylic acid and its derivatives	2.35	Up
	D-Arabinono-1,4-lactone	Organic compound	2.22	Up
	Cinnamyl alcohol	Phenylpropanoid	1.69	Up
	Glutamic acid	Carboxylic acid and its derivatives	0.2	Down
Y10 vs. Y5	5-Chloro-3-methylcatechol	Else	52.32	Up
	4-Hydroxyamphetamine	Benzene and its derivatives	31.47	Up
	4-Methylbenzyl alcohol	Benzene and its derivatives	25.32	Up
	3-Methylxanthine	Fatty acyl	13.4	Up
	2-Thiophenecarboxaldehyde	Organic compound	10.97	Up
	Salicylic acid	Benzene and its derivatives	6.29	Up
	Aminobutyric acid	Carboxylic acid and its derivatives	0.28	Down
	3-methyl-1-butylamine	Organic Nitrides	0.07	Down
Y15 vs. Y10	3-(4-Methylphenyl)-2-propene	Phenylpropanoid	11.42	Up
	Diethanolamine	Organic Nitrides	0.22	Up
	Hydroxyisocaproic acid	Fatty acyl	1.57	Up
	D-Arabinitol	Organic compound	0.6	Down
	norpsitropine	Alkaloid	0.53	Down
	L-Serine	Carboxylic acid and its derivatives	0.62	Down
	Homoserine	Carboxylic acid and its derivatives	0.32	Down
Y20 vs. Y15	4-Guanidinobutyric acid	Carboxylic acid and its derivatives	11.89	Up
	Pentadecanoic acid	Fatty acyl	3.6	Up
	Acrylphenol	Benzene and its derivatives	1.84	Up
	L-Valine	Carboxylic acid and its derivatives	1.81	Up
	Hydroxyisocaproic acid	Fatty acyl	0.55	Down
	Rimycin	Else	0.29	Down
	Acetate	Isopentenol lipids	0.15	Down
Y25 vs. Y20	Myrtenic Acid	Else	0.66	Down
	1H-Imidazole-1-acetic acid	Carboxylic acid and its derivatives	0.16	Down
	4-Methylaminobutyric acid	Else	0.17	Down
	Methyl 2-hydroxybenzoate	Benzene and its derivatives	0.24	Down
	2-Thiophenecarboxaldehyde	Organic compound	0.39	Down
	Salicylic acid	Benzene and its derivatives	3.57	Up
	Trihydroxytoluene	Else	1.64	Up
Y30 vs. Y25	Dihydroxyacetone phosphate	Organic compound	4.75	Up
	Diisopropyl phosphate	Organic phosphoric acid and its derivatives	4.54	Up
	P-Mentha-1.8-Dien-10-yl	Terpenoid	2.01	Up
	3-Hexenyl 2-methylbutyrate	Fatty acyl	1.59	Up
	2-Ethylidene aldehyde	Organic compound	1.80	Up
	5-Isopropyl-2-2-cyclohexen-1-one	Organic phosphoric acid and its derivatives	0.52	Down
	1,2-Dihydroxycyclohexa	Else	0.62	Down
Y35 vs. Y30	Hippuric acid	Benzene and its derivatives	36.86	Up
	Hydroxypyruvic acid phosphate	organic compound	2.67	Up
	β-Alanine	Carboxylic acid and its derivatives	0.13	Down
	L-Ornithine	Carboxylic acid and its derivatives	0.18	Down
	Diphenylamine	Benzene and its derivatives	0.52	Down
	Ethyl 3-phenylpropionate	Fatty acyl	0.40	Down

## Data Availability

The original contributions presented in the study are included in the article. Further inquiries can be directed to the corresponding authors.

## Abbreviations

The following abbreviations are used in this manuscript:

POV	Peroxide value
MDA	Malondialdehyde
CDV	Conjugated diene value
COV	Carbonyl value
PBN	N-tert-butyl-α-phenylnitrone
GC	Gas chromatography
GC–MS	Gas chromatography–mass spectrometry
EI	Electron impact
QC	Quality control
UPLC	Ultra-performance liquid chromatography
ESI	Electrospray ionization
PUFAs	Polyunsaturated fatty acids
UFAs	Unsaturated fatty acids
MUFAs	Monounsaturated fatty acids
SFAs	Saturated fatty acids
BPC	Base peak chromatogram
PCA	Principal component analysis
HCA	Hierarchical cluster analysis
FC	Fold change
OPLS-DA	Orthogonal partial least squares–discriminant analysis
VIP	Variable importance in projection
TBA	Thiobarbituric acid
TEP	1,1,3,3-Tetraethoxypropane
DNPH	2,4-Dinitrophenylhydrazine
MSI	Metabolomics standards initiative
FDR	False discovery rate
